# Supplementary material for: Transcriptomic analysis of potato (Solanum tuberosum L.) tuber development reveals new insights into starch biosynthesis
Source: PLoS One. 2024 Apr 4;19(4):e0297334. doi: 10.1371/journal.pone.0297334 (PMC10994339; doi:10.1371/journal.pone.0297334)
Supplement: Attachment — Submitted filename: Response to reviewers_Shirani Plos one.docx [file pone.0297334.s002.docx]

Dear Editor

Hello

First of all, the authors would like to thank you for giving us the opportunity to present our work to Plos one Journal. We are very excited to have been given the opportunity to revise our manuscript. We want also to appreciate the editorial office for taking the time and effort necessary to provide such insightful guidance. Second, our sincere gratitude to the respected reviewers for their very thorough review, the constructive criticisms, their insightful comments and for helpful suggestions which definitely helped us to improve the presentation of our paper. We cross our finger and hope that these revisions have improved our paper such that you and the respected reviewers now consider it worthy of publication in Plos one Journal. For detail, please refer to the comment by comment responses to the reviewers. Revisions required by reviewer #1 and reviewer #2 have been highlighted **in yellow and in green** in the manuscript, respectively

Yours faithfully

Prof. Farhad Nazarian-Firouzabadi

Corresponding Author

Reviewer #1: The article entitled “Transcriptomic analysis of potato (Solanum tuberosum.) tuber development reveals new insights into starch biosynthesis” presents the interesting study on potato tuber development and starch biosynthesis. The article is generally well-written and organized, presenting the methodology, results, and implications of the study in a clear and concise manner. The article will make a valuable contribution to the scientific literature in this field. However, I recommend to address the following comments and resubmit.

Authors’s Response: We are grateful to the respected reviewer for his/her gentle compliment as well as constructive comments.

Comment 1: Line 13-15 can be rewritten as: "Many genes play major roles in different pathways, including carbohydrate metabolism during the potato tuber's life cycle," to increase the readability of the sentence.

Authors’s Response: Revised accordingly.

Comment 2: In line 97, mention the pot mixture and whether it will influence tuber formation.

Authors’s Response: Characteristics of the Marfona cultivar and soil type used for planting have been added to the manuscript.

Comment 3: In line 98, mention the year in which the experiment was carried out. Also, mention how the light and dark conditions were maintained.

Authors’s Response: Experiment was conducted in 2016. Maintenance conditions have already been mentioned in Line 102-103.

Comment 4: In line 100, mention the days after planting at which samples are taken for the study. Also, mention the size of the tubers.

Authors’s Response: Sampling was done 6 weeks after planting. The size and the shape of samples were according to the description in the material and methods (figure 1).

Comment 5: Mention the agronomic performance of the cultivar and why a particular cultivar was preferred for the study.

Authors’s Response: Marfona cultivar is a popular early baker, high-yielding, resistance to dry rot, and susceptible to potato cyst nematode that is cultivated worldwide. Characteristics of the Marfona cultivar have been added to the text.

Comment 6: In line 107, provide the company name/instrument name instead of the country.

Authors’s Response: Novogene Bioinformatics Technology is a company based in Beijing, China

Comment 7: Mention whether the statistical enrichment of DEGs was tested. If tested, mention the software used.

Authors’s Response: Thank you for the comment. DEGs are the output of Deseq2 package. As mentioned in the manuscript, The DESeq2 package calculate differential expression based on the negative binomial distribution model which is a strong statistical package based on R-language. By default, Deseq2 uses the Wald test (a chi-square test).

Comment 8: Mention whether the starch content of the potato tuber was analyzed at different stages.

Authors’s Response: Appreciate for the comment. Unfortunately, we did not analyzed the starch content of developing tubers due to small size of tuber stages. We needed a delicate/sensitive kit in order to measure the small amount of starch present in tiny tubers. Unfortunately measuring carbohydrates with available manual protocols did not give us an accurate estimate of tiny tubers starch content.

Comment 9: In line 172-174, rearrange the sentence to read: "635 genes, followed by 315 genes, and then 234 genes."

Authors’s Response: We appreciate. Revised accordingly.

Comment 10: All the protocols and software used should be mentioned only in the Materials and Methods section, not in the Results and Discussion.

Authors’s Response: Appreciate, redundant methods and softwares were deleted from the results and discussion sections.

Comment 11: Mention a few important DEGs identified in the study and their role in tuber formation.

Authors’s Response: We have already mentioned and discussed DEGs associated with starch biosynthesis in results and discussion sections.

Comment 12: Figures were given in the Microsoft PowerPoint presentation. It disrupts the quality of the figures. Hence, please provide them in JPEG or PNG format according to the journal's requirements.

Authors’s Response: Appreciate, we will try whether it can be possible to submit figure individually.

Comment 13: Figure 1 is illegible. Please consider improving the quality for better readability.

Authors’s Response: We tried to enhance the quality of photos taken from tuber developmental stages. We think the quality is now acceptable. I wish we had taken the pictures with professional cameras, with a great regret we did not have one!!

Comment 14: Even though the manuscript is grammatically sound, the complexity of a few sentences may puzzle readers. Hence, consider using simpler sentences to attract more readers.

Authors’s Response: We are grateful to the respected reviewer. We had asked a native speaker to read the manuscript. We went through the manuscript and fixed some of the typo/grammar mistakes.

Reviewer #2: Introduction
The introduction prepared well and the recent literatures were cited
Lno; 55-57 include proper citations

Authors’s Response: Appreciate, appropriate citations were added.

Methodology
The methodology lacks many technical information and need to enhance the English
Mention year and station of the experiment conducted

Authors’s Response: Appreciate, Revised accordingly.

Lno 105; rna quality checked in gel or nanodrop, mention properly

Authors’s Response: Ambiguity was fixed. The new sentence meaning that the quality and quantity of RNA was double checked by both methods.

Lno 109; how 18 samples selected and what are all the six samples, is there any replication means mention properly

Authors’s Response: RNA samples were selected for sequencing based on RIN number (RIN>7). Details have been incorporated in the manuscript.

Lno 114; is it Trimmomatic version 3-6 or 3.6 ?

Authors’s Response: Appreciate, we used Trimmomatic version 0.36 .

Lno123: what are all the different databases like NCBI nr, uniprot, etc
Concise the RT PCR portions

Authors’s Response: We used Go and NCBI non-redundant databases. RT-qPCR details have been already mentioned in Line 153-155.

Results
Lno 156: method and software should not repeat here.

Authors’s Response: Appreciate, redundant methods and softwares were deleted from the results section.

Give raw reads and filtered reads and the statistics table about the rnaseq reads The obtained results not clearly explained and mainly not able to understand the concept Need to improve the manuscript results section with proper English editing

Authors’s Response:We are most grateful to the respected reviewer. We have now added table 1, showing the summary of read numbers based on the RNA-Seq data analysis from developing potato tuber stages.

Discussions
Well discussed with previous reports

Authors’s Response: Our best greetings for respected reviewer nice compliment.

Figures
Include significance in Fig 6 can try with the better visualization graphs like volcano plot, kegg and gene ontology bar graphs it helps to better understanding for readers.

Authors’s Response: Expressions were compared with student t-test relative to SI. The figure 6 graph has now been modified with statistics signs showing the differences. We agree with the respected reviewer to use volcano plot, however, since the number of genes investigated are small, volcano plot does not look informative.
