# Supplementary material for: Transcriptomic analysis of potato (Solanum tuberosum L.) tuber development reveals new insights into starch biosynthesis
Source: PLoS One. 2024 Apr 4;19(4):e0297334. doi: 10.1371/journal.pone.0297334 (PMC10994339; doi:10.1371/journal.pone.0297334)
Supplement: Attachment — Submitted filename: Response to reviewers.docx [file pone.0297334.s003.docx]

Dear Editor

Happy new year and wishing you all the best for 2024. I hope that this year bring you lots of happiness and joy.

We have dealt with the respected academic editor and reviewers’ comments. A native speaker has also revised our manuscript and proofreading can be seen in the revised version of the manuscript. We hope that our manuscript is now good enough to be published in the Plos one journal.

Yours truly

**ACADEMIC EDITOR:** I suggest following the reviewer's suggestions and modifying the manuscript. Also, I recommend improving the english language.

Author’s Response: Thank you so much for your suggestion. We had our manuscript revised by a native speakers.

Reviewer #1:
I hope this message finds you well .I appreciate the valuable contribution your research. While the content of your article is robust and insightful, I have noted a few areas where attention to English language usage and clarity could enhance the overall quality of the manuscript. Please consider removing the words like "generally speaking".

Author’s Response: we appreciate the respected reviewer comments. We had our manuscript revised by a native speakers.

I observed that there seems to be an overlap in the presentation of materials and methods, specifically pertaining to software, in both the Materials and Methods section and the Results section. To ensure clarity and adherence to the conventional structure of scientific manuscripts, it is recommended to confine details regarding materials, including software, strictly to the Materials and Methods section.

Author’s Response: Thank you so much for your constructing comment. We omitted the overlapping material and methods details from the results section.

Kindly consider revisiting the Results section and refraining from introducing new materials and methods information. If any software or specific methods are crucial to understanding the results, it would be best to reference the earlier Materials and Methods section where they are appropriately detailed.

Author’s Response: We went through the result section and deleted the unnecessary material and methods details.
